# Process of Disinfection

**Published:** 1865-10

**Authors:** 


### PROCESS OF DISINFECTION.

The following memoranda on disinfection have just been issued by the Privy Council; and, considering the circumstances under which they are published, we feel bound to assist in giving them all publicity:

"1. For purposes of artificial disinfection, the agents which most commonly prove useful are—chloride of lime, quicklime, and Condy's manganic compounds. Metallic salts—especially perchloride of iron, sulphate of iron, and chloride of zinc, are, under some circumstances, applicable. In certain cases chlorine gas or sulphurous acid gas may advantageously be used; and, in certain other cases, powdered charcoal or fresh earth.

"2. If perchloride of iron or chloride of zinc be used, the common concentrated solution may be diluted with eight or ten times its bulk of water. Sulphate of iron or chloride of lime may be used in the proportion of a pound to a gallon of water, taking care that the water completely dissolves the sulphate of iron, or has the chloride of lime thoroughly mixed with it. Condy's stronger fluid (red) may be diluted with fifty times its bulk of water; his weaker fluid (green) with thirty times its bulk of water. Where the matters requiring to be disinfected are matters having an offensive smell, the disinfectant should be used till this smell has entirely ceased.

"3. In the ordinary emptying of privies or cesspools, use may be made of perchloride of iron, or chloride of zinc, or of sulphate of iron. But where disease is present, it is best to use chloride of lime or Condy's fluid. Where it is desirable to disinfect, before throwing away the evacuations from the bowels of persons suffering from certain diseases, the disinfectant should be put into the night-stool or bed-pan when about to be used by the patient.

"4. Heaps of manure or other filth, if it be impossible or inexpedient to remove them, should be covered to the depth of two or three inches with a layer of freshly burnt vegetable charcoal in powder. Freshly burnt lime may be used in the same way, but is less effectual than charcoal. If neither charcoal nor lime be at hand, the filth should be covered with a layer some inches thick of clean dry earth.

"5. Earth, near dwellings, if it has become offensive or foul by the soakage of decaying animal or vegetable matter, should be treated on the same plan.

"6. Drains and ditches are best treated with chloride of lime, or with Condy's fluid, or with perchloride of iron. A pound of good chloride of lime will generally well suffice to disinfect 1,000 gallons of running sewerage; but, of course, the quantity of disinfectant required will depend upon the amount of filth in the fluid to be disinfected.

"7. Linen and washing appearel requiring to be disinfected should without delay be set to soak in water containing per gallon about an ounce either of chloride of lime or of Condy's red fluid. The latter, as not being corrosive, is preferable. Or the articles in question may be plunged at once into boiling water, and afterwards when at wash be actually boiled in the washing water.

"8. Woolens, bedding, or clothing which cannot be washed, may be disinfected by exposure for two or more hours in chambers constructed for the purpose to a temperature of 210 to 250 degrees Fahrenheit.

"9. For the disinfection of interiors of houses, the ceilings and walls should be washed with quicklime water. The woodwork should be well cleansed with soap and water, and subsequently washed with a solution of chloride of lime, about two ounces to the gallon.

"10. A room, no longer occupied, may be disinfected by sulphurous acid gas or chlorine gas—the first by burning in the room an ounce or two of flowers of sulphur in a pipkin; the second, by setting in the room a dish containing a quarter of a pound of finely powdered black oxyd of manganese, over which is poured half-a-pint of muriatic acid, previously mixed with a quarter of a pint of water. In either case the doors, chimney, and windows of the room must be kept carefully closed during the process, which lasts for several hours.—*Chemical News.*

